# Conversing with a devil’s advocate: Interpersonal coordination in deception and disagreement

**DOI:** 10.1371/journal.pone.0178140

**Published:** 2017-06-02

**Authors:** Nicholas D. Duran, Riccardo Fusaroli

**Affiliations:** 1 School of Social and Behavioral Sciences, Arizona State University, Glendale, Arizona, United States of America; 2 The Interacting Minds Centre, Aarhus University, Aarhus, Denmark; Universita degli Studi di Verona, ITALY

## Abstract

This study investigates the presence of dynamical patterns of interpersonal coordination in extended deceptive conversations across multimodal channels of behavior. Using a novel "devil’s advocate" paradigm, we experimentally elicited deception and truth across topics in which conversational partners either agreed or disagreed, and where one partner was surreptitiously asked to argue an opinion opposite of what he or she really believed. We focus on interpersonal coordination as an emergent behavioral signal that captures interdependencies between conversational partners, both as the coupling of head movements over the span of milliseconds, measured via a windowed lagged cross correlation (WLCC) technique, and more global temporal dependencies across speech rate, using cross recurrence quantification analysis (CRQA). Moreover, we considered how interpersonal coordination might be shaped by strategic, adaptive conversational goals associated with deception. We found that deceptive conversations displayed more structured speech rate and higher head movement coordination, the latter with a peak in deceptive disagreement conversations. Together the results allow us to posit an adaptive account, whereby interpersonal coordination is not beholden to any single functional explanation, but can strategically adapt to diverse conversational demands.

## Introduction

From bold-faced lies to more benign fibs, deception is interwoven in social life. The goal of deception is to convince others of sincerity while communicating information known to be false while also avoiding detection. In an everyday conversational context, deceivers can hide behind communicative assumptions of cooperation, relevance, and honesty [1–2], as well as behind the complexity and rapid changes characterizing face-to-face conversation [3]. Attentional and cognitive resources are often limited, and in such complex contexts, one might argue naïve partners to be less likely to explicitly notice a duplicitous interlocutor’s incriminating behaviors. Nevertheless, would-be deceivers should take pause. There are a number of cognitive challenges in creating and maintaining deception during social interaction that are subtly manifested through subtle dynamics in the verbal and nonverbal channels of behavior [4–6]. Recent research has made significant progress in exposing these otherwise hidden behaviors through the use of automated tools that bypass the need for human judgments and that target patterns of low-level and time-evolving sequences of behavior [7–9].

Great strides have also been made in understanding the deceiver-level changes that occur in conversation. It has become increasingly clear that contextual and partner-specific factors are of particular importance in shaping how deceivers behave [10–12]. However, interpersonal interactions are constituted by more than the isolated behaviors of the interlocutors. Interlocutors spontaneously entrain their bodily movement, breathing and turn-taking, re-use each words, and develop shared routines as they interact [13]. Interpersonal behavioral coordination has been found to be ubiquitous and has been argued to serve a range of communicative functions, such as forging shared understanding and creating a sense of social rapport [14–16]. Nevertheless, little empirical research has been done on whether less savory contexts, such as deceptive and conflictual interactions, might also involve and modulate behavioral coordination.

### Current approach

The goal of this paper is to bridge this gap by systematically investigating interpersonal behavioral coordination within naturalistic open-ended deceptive and truthful conversations. We examine conversational deception across unscripted extended conversations where varied, but experimentally controlled, goals exist. To this end, we introduce a novel “devil’s advocate” paradigm that selectively elicits deception (having to deceptively argue for a position opposite to what a participant actually believes) while also manipulating whether participants agree or disagree with each other (having to argue for a position a conversational partner does or does not share). In these interactions, deceivers’ partners are also naïve to the possibility of deception and both partners are as unrestricted as possible in what is said. This is a divergence from previous deception research where a small number of potential partners delivered instructions in a prescribed manner, and in which the deceiver knows that the partner is looking for signs of guilt. Although this can have notable advantages in terms of content control and simulating forensic contexts, it potentially limits the dynamical mechanisms that are central to the emergence of shared signals in open-ended and unconstrained conversations—the very contexts in which everyday deception predominantly occurs [17].

We situate our work within a synergistic view of behavioral coordination [3, 18]. According to the synergistic approach, the complexity of interpersonal interactions is tackled by reducing the degrees of freedom of the interlocutors’ behaviors, that is, by making them interdependent on each other. In this way, the analysis is conducted at the level of the dyad and not reduced to the behavior of a single participant (c.f., [19–21]) Across many modalities (from postural way to lexical choices), people entrain their behaviors to each other: synchronizing their rhythms, fine-tuning their turn-taking, aligning the actual behaviors, and even assuming complementary roles. Coordinated behaviors do not need to be isomorphic and occur close in time, as would be the case for behavioral mimicry [14]; rather, they can be distributed and loosely coupled across various local and global temporal scales [22–25]. The analysis of such coordination requires the use of unique statistical methods that capture time-evolving interdependent behaviors, including windowed lagged cross correlation and cross recurrence quantification analysis, two methods employed in the current study. Crucially, the synergistic approach argues that temporal patterns of low-level, continuous, and spontaneous behavioral coordination work in concert with more intentional higher-level processes, so that behavioral coordination is shaped by the goals and context of the interaction (c.f., [26]). We thus evaluate the coordination of complementary behavioral channels—continuous head movements and speech rate—across different conversational contexts.

Head movements between conversational partners tend to nonlinearly interact in closely aligned windows of time and are amenable to evaluation as locally coupled sequences of shared activity across minimal delays in time. Speech rate is a complementary measure to movement in that it allows us to examine the more global properties of coordination. Speech occurs in largely non-overlapping turns between interlocutors: when one interlocutor speaks, most often the other does not. Moreover, coordination of speech rate does not necessarily have to occur over contiguous sequences, but can have temporally extended influences. Increases of speech rate by one partner early in a conversation can be echoed by the interlocutor later in the conversation. Additionally, we expect both behaviors, head movements and speech rate, to become coordinated due to their importance in signaling communicative functions of shared attention, active participation, and cooperation [27–30]. Indeed, there is a close relationship between head movement coordination and conversational outcomes [31–32], including in contexts involving disagreement/agreement [33] and deception [34]. A great deal of research has also shown that people unintentionally align and coordinate their speech during communication [35–37], where doing so serves to index feelings of closeness and attitude similarity [38–40].

### Hypothesized patterns of coordination

The focus on examining deception during disagreement and agreement conversations allows us to explore how behavioral coordination might change as a result of a change in high-level conversational goals. Typically, interpersonal behavioral coordination is thought to enable shared mental and action representations [41, 15], as well as index general positive social outcomes, such as increased liking and rapport, blurred self-other boundaries, and enhanced altruistic behavior and cooperation [16, 42–43]. It is assumed that when these shared informational and affiliative processes are disrupted, as in disagreement, decreased and less stable behavioral coordination will follow [44–45]. Thus, we predict that when deception is not a factor, agreement will show greater behavioral coordination than disagreement conversations.

It becomes more of an open question as to how behavioral coordination will be expressed when deception is introduced. At one level, deceivers must continue the normal work of collaborating with their conversational partners to establish shared meaning, but at the same time, they have to navigate a number of cognitive challenges associated with deception: i.e., the inhibition of a truth bias [46–47], cognitive control in delimiting truth from lies [48], and the generation of imagined events [49]. As a consequence, deception, like disagreement, can be thought of as being disruptive. Moreover, in a situation where a conversation involves both deception and disagreement, a situation of maximum disruption, behavioral coordination might be most impaired.

But there is also an alternative hypothesis to consider—one based on the aforementioned synergistic view. Rather than being an indiscriminate index of cognitive load or rapport, behavioral coordination may instead arise from strategic, *adaptive* conversational goals that override these factors [3, 18]. From this perspective, behavioral coordination serves multiple functions that depend on unique contextual demands. In deception, a particularly important demand, at least for the deceiver, is in managing appearances of believability to avoid violations of social norms [50]. To do so requires increased vigilance and attentiveness in responding to a partner’s behavior [11, 51]. In turn, this greater attunement to the other, particularly in extended interpersonal interaction marked by an open channel of reciprocal involvement, could result in increased and more stable coordination.

Conversations involving deception and disagreement also raise unique possibilities in that, because this situation is one in which the goal of believability is most threatened, deceivers must be particularly attuned to their partners’ behaviors. As a result, behavioral coordination in these conversations, rather than being most impaired, will instead be most pronounced. There is some support for this prediction based on recent work by [52]. In their study, behavioral coordination between deceivers and a confederate partner was assessed by a group of human raters who rated, amongst other measures of behavior, a general "gestalt" of perceived synchrony. Critically, impressions of behavioral coordination were highest in deception during a conflictual versus neutral phase of the interaction. Although the conditions of [52] vary greater from our current approach, it does open up the possibility that behavioral coordination during deception and disagreement/conflict will also be pronounced in the current analyses.

## Paradigm and participants

### Devil’s advocate

Participants were recruited to have two 8-minute conversations about political and social topics that typically engender strong opinions. Written informed consent was obtained from every participant prior to the study, and the procedure was approved by the local Ethics Review Board (University of California Merced). All participants were compensated with extra course credit for participation.

Participants were led to separate private rooms where they completed a 10-item questionnaire developed by [45]. These items required participants to provide a one- to two-sentence rationale to support their true opinion on abortion, universal health care, gay marriage, marijuana legalization, death penalty, political party affiliation, war in the middle east, legal drinking age, taxing rich Americans, and financial aid criteria. For each item, the strength of their opinion was also recorded on a 4-point Likert scale. The responses were used to optimally select topics that participants agreed or disagreed on (e.g, either a score of “4” for “feel very strongly,” and if not available, “3” for “feel somewhat strongly”). In the rare occasions where this selection criterion could not be met, participants were excused from further participation. The topics assigned for each conversation, for each dyad, can be found at: https://github.com/nickduran/coordination-deception.

Participants were then led to a large common room where they stood face-to-face at a distance of approximately 6 feet (and no less than 3 feet given bounded regions marked on the floor). The experimenter provided instructions depending on the experimental conditions for the conversation: agreement vs. disagreement (a between-subjects manipulation) and devil’s advocate vs. honest conversation (a within-subjects manipulation). Importantly, the order in which the devil’s advocate and honest conversations occurred was counterbalanced between participants. If the participants were to start with a devil’s advocate conversation, they were asked to go back to separate rooms to complete a new set of questionnaires. One participant was randomly selected to act as the devil’s advocate (hereafter referred to as DA). The other participant (hereafter "naive") was given a short questionnaire to assess general emotional state and was asked to wait in the room until the experimenter returned. The naive participant was also told that a problem with the audio equipment had to be addressed and the wait might be a few minutes.

The experimenter then entered the private room of the DA, who was informed that she had been selected to discuss a topic with the naive participant by taking an opinion opposite of her own true beliefs. In the disagreement condition, we chose the topic in which participants had originally shared a similar opinion. For example, if the DA supported marijuana legalization, she had to now argue against legalization with a partner who truly supported it. In this way, the conversation involved ostensible disagreement with one partner providing information that is known to be false to a partner who is unaware of the true state of affairs. Conversely, in the agreement condition, we chose the topic in which participants had originally given dissimilar opinions.

Instructions were also given to the DA not to reveal her actual beliefs to the naive participant (thus, the DA was instructed to lie), and that lying successfully in this way is indicative of skilled argumentative abilities. The DA was given three minutes to consider various talking points to be used in the upcoming conversation. Participants were then brought back into the main common room and again stood face-to-face at a comfortable distance. At this point both participants were told which topic they would discuss (e.g., marijuana legalization). For those in the disagreement condition, participants were also told that they should attempt to convince the other of their opinion. For those in the agreement condition, participants were told that they should discuss the merits of their shared opinion in order to prepare for a hypothetical debate with a team that holds the opposite view. The importance of staying on topic was also stressed.

After conversing for eight minutes, participants were instructed to return to their original separate rooms to complete a three-item questionnaire to gauge interpersonal rapport after conversing (cf. Rapport/experience" section and [45]). Participants were then brought back into the main common room for a second conversation where they honestly discussed a topic in which both agreed or disagreed depending on their assigned condition. After this conversation, participants returned to separate rooms to complete the questionnaire set and were finally debriefed. Participants were also asked whether they suspected anything unusual about the conversations. No naive participant reported suspicion of deception.

### Participants

We recruited 116 undergraduate students through a university’s recruitment website over the course of 6 months, from May to November 2012. Written informed consent was obtained from every participant prior to the study, who were also aware that video and audio recordings of their behavior would be collected and thus they were not completely anonymous to researchers during and after data collection. All procedures were approved by the local Ethics Review Board. All participants were compensated with extra course credit for participation. Twelve pairs had to be discarded due to technical problems (e.g. poor or absent audio) or instructions not being followed (e.g. openly revealing the deceptive stance). This resulted in 22 pairs for the disagreement, and 24 for the agreement condition. The final number of dyads analyzed (46) is similar to other studies conducted in this area (e.g., 32 in [11]; 24 in [53]; 32 in [45]; 21 in [54]). Dyads were largely mixed-sex and female (mixed: 20; female-female: 22; male-male: 4). No dyads reported knowing each other well prior to the interaction. A detailed account of age, gender, and ethnicity of each participant can be found at: https://github.com/nickduran/coordination-deception.

## Measurement and quantification

During each interaction, the speech and movements of participants were recorded with lapel microphones attached to each participant and connected to a Canon HD Vixia camcorder. The camcorder was placed approximately 15 feet from the participants to capture a side view of the interaction. A side view provides a rich signal of continuous movements between participants with a camera that is not directly in participants’ line of sight. Audio was recorded as separate channels and synched to the video. The video and audio streams were analyzed using automated computational techniques, capturing a time series of head movements and speech rate. We then compared the time series from each dyad, for each modality, to derive modality-specific measures of interpersonal coordination.

### Capturing individuals’ movements

**Fig 1:**
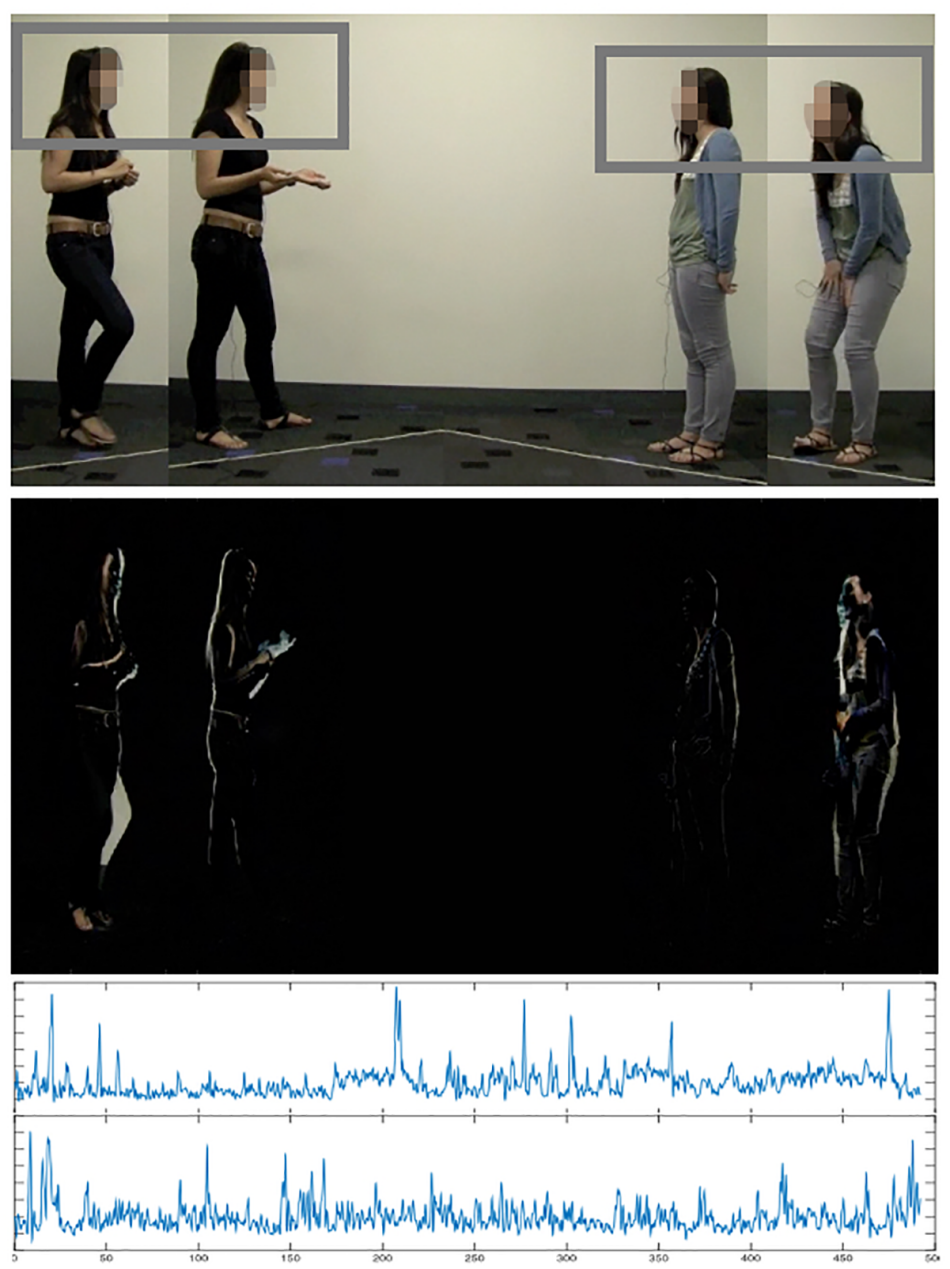
Extracting continuous movement displacement from video recordings of individuals’ movements. (Top panel) Example of movement change across two sequential points in time, targeting head movements (gray boxes). (Middle Panel) Pixels that change from frame-to-frame are converted to a white dot for visualization purposes, producing motion energy flows. (Bottom panel) The number of pixels that change from frame-to-frame, repeated over the length of the video, are converted into a time series that captures degree of movement displacement for each participant.

We isolated participants’ undifferentiated head movements (used in the main analyses) and lower body movements (used as a control, as explained in the analysis) from the video recordings of their conversational interactions. These movements are undifferentiated in that, rather than head movements as isolated and discrete events, they are treated as a continuous, gestalt-like signal of rhythmic change, also known as *motion energy flows* [55]. These are extracted with a technique similar to that of [55], and recently implemented by [56] (but extended here to focus on targeted body regions). Using a custom-made program developed in the Matlab computing language and available at https://github.com/nickduran/coordination-deception, conversational videos, sized at 640x360 pixels, and shot at 30 frames per second, were processed using what is called a frame subtraction method. This method takes advantage of the RGB values encoded by each pixel (three color values, ranging from 0 to 255) in each video frame. When people move, RGB values will change from frame to frame across corresponding pixels and remain static for pixels capturing the background. As movements become more pronounced, more pixels will be affected. We then compute the absolute summed difference across the pixel changes across every 5th consecutive frame (i.e., a sampling rate of 6Hz). This is plotted as a time series where the y-axis represents the absolute summed difference values (please see Fig 1 for a systematic walk-through of this method). The result is a continuous signal of movement displacement that captures the duration and extent of movement change.

#### Quantifying (tightly coupled) coordination in the movement signals

In the next step, we apply a windowed lagged cross correlation (WLCC) technique to derive measurements of shared behaviors. WLCC has recently been used in a number of analyses to assess how two time series change together on a moment-by-moment basis [25, 34], and is robust against statistical assumptions that are problematic for more traditional analyses, such as assumptions of stationarity (i.e. that mean and standard deviation do not change over time). WLCC computes cross correlations within small moving windows of time (ten seconds), and then aggregates these windows to produce an overall measure of similarity. Furthermore, synchrony between participants at different points in time can be compared by lagging one partner’s time series relative to the other (at increments of 1/6 of a second up to 5000ms), and then repeating the process of aggregating across temporal windows. These aggregated points are then plotted, producing, for example, the WLCC profiles reported in the Results section.

The first measure we derive, hereafter referred to as "~Lag 0ms," is based on cross correlations that include lag 0ms and one time step on either side of 0ms. This region corresponds to near-simultaneous shared activity. The second and third measures capture the average cross correlation values for all lags outside ~Lag 0ms, up to 1000ms. Each of these lags corresponds to the immediate responsiveness of one partner relative to the other. For example, the cross correlation at a positive lag of 800ms is how similar the DA’s movements were to what the naive participant was doing 800ms earlier (DA following). Likewise, the cross correlation at a negative lag of 400ms is how similar the naive’s movements were to what the DA was doing 400ms earlier (Naive following). Given that the positive and negative lags correspond to differences in who follows who, the measurement over positive lags is hereafter referred to as "DAFollows 1000ms" and the measurement over negative lags is hereafter referred to as "NaiveFollows 1000ms."

### Capturing individuals’ speech rate

About 608 minutes of open-ended conversational dialogue were collected: two eight-minute conversations from each of the 46 dyads. Each speaker’s audio-channel was preprocessed to remove low-frequency background noise. Within the Praat environment—a widely used speech analysis computer program [57]—a team of four human "taggers" were trained to mark the beginning and end of each spoken utterance by the use of auditory and visual cues (e.g., audio playback features, onset and offset of energy peaks in the waveform). The manual tags were then adjusted to a 10 milliseconds precision scale through an automated analysis of pitch presence/absence and intensity changes using Matlab (Mathworks Inc.). In cases where separate stereo channels were not available and conversations had to be transcribed across a mono channel, taggers relied mostly on audio feedback (although the single waveform was visible; this situation applied to 12 conversations due to experimenter error in setting up recording equipment).

We employed utterance boundaries to precisely extract fundamental frequency (Hz) and intensity (dB) using Praat and correcting for octave jumps and other artifacts. Voiced peaks in intensity were automatically isolated and employed as proxies for vowel onsets, according to the procedure in [58]. In order to assess shared dynamics between the interlocutors, we could not rely on a simple count of average syllable count per minute, we instead needed a continuous time series displaying changes of pace over time. Therefore, we used 5-second windows with a 333ms slide to generate time-series of estimated syllables per minute at 3Hz. In other words, we estimated how many syllables would have been generated if the speaker had maintained that rhythm for a full minute (multiplying the number of syllables in the 5 second window by 20). Then we shifted the window forward of 333ms and repeated. This procedure was validated for other interval time series in [59]. We thus achieved continuous uniformly sampled time-series of estimated syllables per minute, analogous to the movement displacement time-series.

#### Quantifying (global) coordination in the speech rate signals

To derive measures of global coordination, we cannot use WLCC because it requires the events analyzed to co-occur in time or at fixed lags. Instead, we employed Cross Recurrence Quantification Analysis (CRQA), a nonlinear and more flexible analog of cross correlation that quantifies shared dynamics between time series. CRQA employs the Takens theorem [60] to reconstruct the phase space in which the two time series move. In other words, CRQA identifies all possible combination of states in which the two time series can be (e.g. A speaking 4 syllables per second, while B 5 per second; and on to all other possible combinations of values). CRQA then maps the trajectory of the time series at all possible lags within such phase space, isolating those instances in which the two speakers present similar speech rate dynamics. By reconstructing the possible states of the two systems and assessing the points in time in which they visit similar states, CRQA quantifies how often the two systems display similar patterns of change, and how complex the structure of the entrainment between their trajectories is. This analysis of entrainment across all possible lags enabled us to analyze coordination in speech rate time series that presented a turn-taking structure. CRQA was originally designed to explore how two systems come to share similar dynamics in a common state space. It has been applied to many types of biological and physical systems, where an earlier state of one signal, as a state attractor, can influence the states of another signal removed in time. In terms of two people talking, the earlier speech rate of one participant can have an influence on the other later in the interaction. CRQA thus assesses coordination that is not necessarily limited to contiguous sequences of behavior, and quantifies its global properties, such as temporal extension and flexibility.

CRQA was then used to assess how similar the general dynamics of speech rate were across interlocutors: do we observe similar values of speech rate across interlocutors? Are sequences of speech rate produced by A re-used later on by B (independently of how much later)? And so on. In particular, CRQA produces different indexes of cross recurrence, which we used to quantify different properties of speech rate coordination:

- *Amount of coordination*: defined as the percentage of single values that mutually recur (are present) across the entirety of both time series (recurrence rate, RR). The higher the amount of coordination, the more the interlocutors will display similar speech rate values, though not necessarily at the same time or displaying the same fine temporal dynamics.
- *Stability of coordination*, articulated in: the percentage of values that do not recur in isolation, but form sequences of contiguous repeated values (DET); average length of sequences repeated across time-series (L); length of longest repeated sequence (LMAX); and average distance between repetitions (T2). The higher the stability of coordination, the more the interlocutors tend to re-use each other’s speech rate patterns, that is, not the single values but structured sequences, and to maintain this coordination for longer stretches of time. As detailed above, CRQA analyzes coordination across all possible time lags, therefore, a stable repeated sequence might involve individual speech rate sequences from distant utterances. What matters is that speech rate values that are contiguous in the first speaker are also contiguous in the second speaker, be that at an earlier or later point.
- *Complexity of coordination*: defined as low if all repeated sequences are of the same length, high if repeated sequences vary in length (entropy, ENTR), thus suggesting that coordination is flexible and not mechanical imitation. The higher the complexity of coordination, the more diversity we observe in the repeated patterns across interlocutors: sometimes the shared sequence is only a 1000ms long, perhaps pertaining to short bouts of backchanneling, sometimes it stretches across much longer periods, as full conversational moves are matched across interlocutors.

These indexes enable us to assess the structure of coordination in terms of whether interlocutors share a similar speech rate, but also in how this similarity is structured in time: just maintaining the same range of values or repeating highly articulated sequences for long stretches of time. All analyses were also calculated using the CRP toolbox in Matlab 2014a. For further details on the methods see [61, 62].

### Additional considerations

#### Virtual pairs

In order to ensure that the levels of coordination observed in movement and speech rate were due to the actual interaction and not simply to the constraints of the task (standing in a room facing another person during a conversation), we compared real pairs of interlocutors with virtual pairs. These virtual pairs were artificially constructed by juxtaposing two interlocutors from different conversations. Doing so breaks up the perceptual and temporal dependencies between partners, but still preserves the general pattern of behavior. If synchrony were found here, this would undermine the claim that coordination emerges from the real-time dynamics of interaction. The virtual pairs control baseline was originally introduced by [63].

#### Rapport/experience

To confirm that our manipulation of agreement and disagreement conversations were perceived by conversational partners as engendering more or less rapport, we asked three questions after each conversation. These questions were worded as declarative statements that could be rated on a Likert-scale ranging from 1 to 6, anchored by the statements “very strongly disagree” to “very strongly agree.” The questions consisted of the statements: a) “I felt very close to my partner,” b) “I felt that my partner understood what I was saying,” and c) “I felt that I understood what my partner was saying.” We also assessed whether the members of a dyad experienced different levels of rapport, or behaved as a system, displaying similar rapport no matter the hidden asymmetry in their roles (one DA and the other naïve). We therefore evaluated the difference score based on the DA’s response subtracted from the Naive partner’s score. A higher absolute difference score would indicate a more divergent opinion, whereas a lower absolute difference score indicates a more shared opinion.

## Analysis

For each dyad, the above procedures generated three movement-based dependent variables (~Lag 0ms, DAFollows 1000ms, and NaiveFollows 1000ms) and six speech-rate based dependent variables (RR, DET, L, LMAX, T2, ENT). These were examined via two diverse analytical approaches. The first is with mixed effect modeling that is best suited for identifying differences while simultaneously taking into account within-participant (in our case, within-dyad) idiosyncrasies. There is a limitation of such modeling in that it remains unclear whether the results might generalize across dyads to any dyad. In other words, if deception involves decreased speech rate, for example, how much do we need to know about a dyad’s baseline speech rate in order to assess the presence of deception? Or is it possible to individuate speech rate thresholds that indicate the general likelihood of deception in any dyad? These are crucial questions for the study of deception and motivate our second analytical approach. We use a cross-validation technique with training and tests sets that assesses the possibility of creating a model of deceptive cues from one set of dyads (training), in order to assess deception in a second set of never-seen-before dyads. These analyses are elaborated upon further in the following two sections.

### Mixed effects models

All comparisons reported here were evaluated using a linear mixed-effects model framework from the lme4 module within the R statistical package [64]. For each model, each index of shared movement dynamics (~0ms, NaiveFollows 1000ms, and DAFollows 1000ms), speech rate (RR, DET, L, LMAX, T2, and ENT), and rapport Likert ratings, were separately used as dependent variable, and the centered factors of Conflict (Disagreement; coded as 0.5 vs. Agreement; coded as -0.5), Veracity (Deceptive; coded as -0.5 vs. Truth; coded as 0.5), Order (whether the deceptive conversation was the first or second of the two conversations) and Sex (Female-Female vs. Female-Male interactions) were entered as fixed-effect predictors. In addition, Dyad (46) and Topic (10) were entered as random effects including random slopes for Conflict and Veracity. Given the limited amount of degrees of freedom in the data, we only looked at interactions between Conflict and Veracity. In cases where the models could not converge due to the complexity of the random effect structure, we removed effects one-by-one until convergence was achieved, simultaneously ensuring via likelihood ratio tests that the simpler model did not statistically vary from the more complex model in terms of variance captured.

For all models we report an overall measure of captured variance, coefficients of the predictors, their standard error, and *p*-values for each of the factors in the model. Captured variance is reported as Marginal R^2^ (Rm^2^)—variance explained by fixed factors alone—and Conditional R^2^ (R^2^)—variance explained by fixed and random factors together—and computed using the MuMIn R statistical package [65]. All statistical code used to generate analyses and de-identified data are available at https://github.com/nickduran/coordination-deception.

### Cross-validation

To find deceptive and disagreement cues that might generalize across dyads, we also assessed the unique contribution of multiple behavioral variables in predicting outcomes of interest, retaining only those that contribute unique sources of variance in making predictions. Thus, if movement and speech channels all reflect the same underlying dynamics, then only a small number of variables is needed.

For these analyses, we employed a 5-fold cross-validated feature selection and multiple regression models [66, 67], using the Statistics, Bioinformatics, and MICP toolboxes in Matlab 2014a. We initially entered 20 potential measures of behavioral synchrony into the model: 14 indexes of motor synchrony (+/- 3000ms at 1000ms intervals for both head and lower body movements), and 6 indexes of speech rate coordination (RR, DET, L, LMAX, ENT, T2). Such a large number of independent variables run the risk of overfitting the data when drawing predictions. To address this, we used a common algorithm to select a parsimonious subset of features tailored to each dependent variable: ElasticNet feature selection [68]. ElasticNet assesses the correlation between variables and selects the minimal subset preserving the overall variance of the dataset. We begin by splitting the dataset into five subsets with each dyad belonging to only one subset. Each subset then becomes the testing set to optimize the features selected by ElasticNet on the other four subsets. Per each dependent variable, we thus employed the relevant optimal variable set in a multiple logistic regression model, maintaining the 5-fold cross-validated procedure. The 5-fold cross-validation ensures generalizability of the results: the model is fit to four fifths of the dyads and the statistical significance of the regression models and their effect sizes are only calculated on the remaining fifth. The statistical accuracy of the logistic regression models was then balanced using variational Bayesian inference, which conservatively compensates for missing data, individual variability along the dyads, and makes sure sensitivity and specificity are at comparable level [69]. Finally, given the random nature of the fold-split, the process was repeated 100 times to assess reliability of the results, reported as mean and confidence intervals across all runs.

## Results: Movement coordination

### Virtual pairs

**Fig 2:**
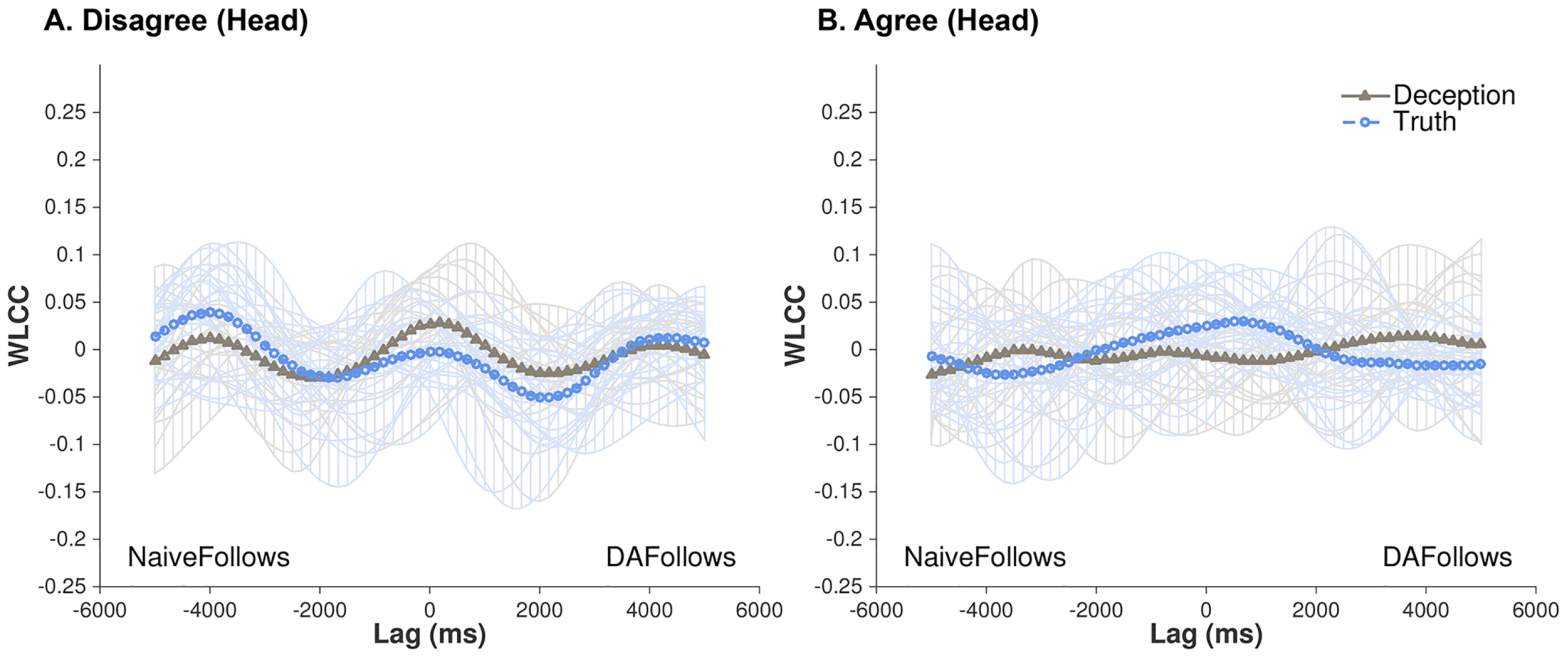
WLCC profiles for DAs and virtual naives’ head movements. (A) Disagree and (B) Agree conversations involving deception (triangle-solid line) or truth (circle-dashed line). No statistically significant effects were found across conditions. Figures also show each dyad’s contributions (opaque lines) to the average pattern across all conditions.

We begin by reporting the virtual pair analysis, where coordination is computed between a DA and a virtual partner taken from another dyad of the same agreement/disagreement condition. As shown in the Fig 2 WLCC profile, there is little visual evidence of coordination between Veracity and Conflict across any lag series, which was statistically confirmed through linear mixed effects models. There were also no statistically significant effects involving Sex or Order.

### Real pairs

**Fig 3:**
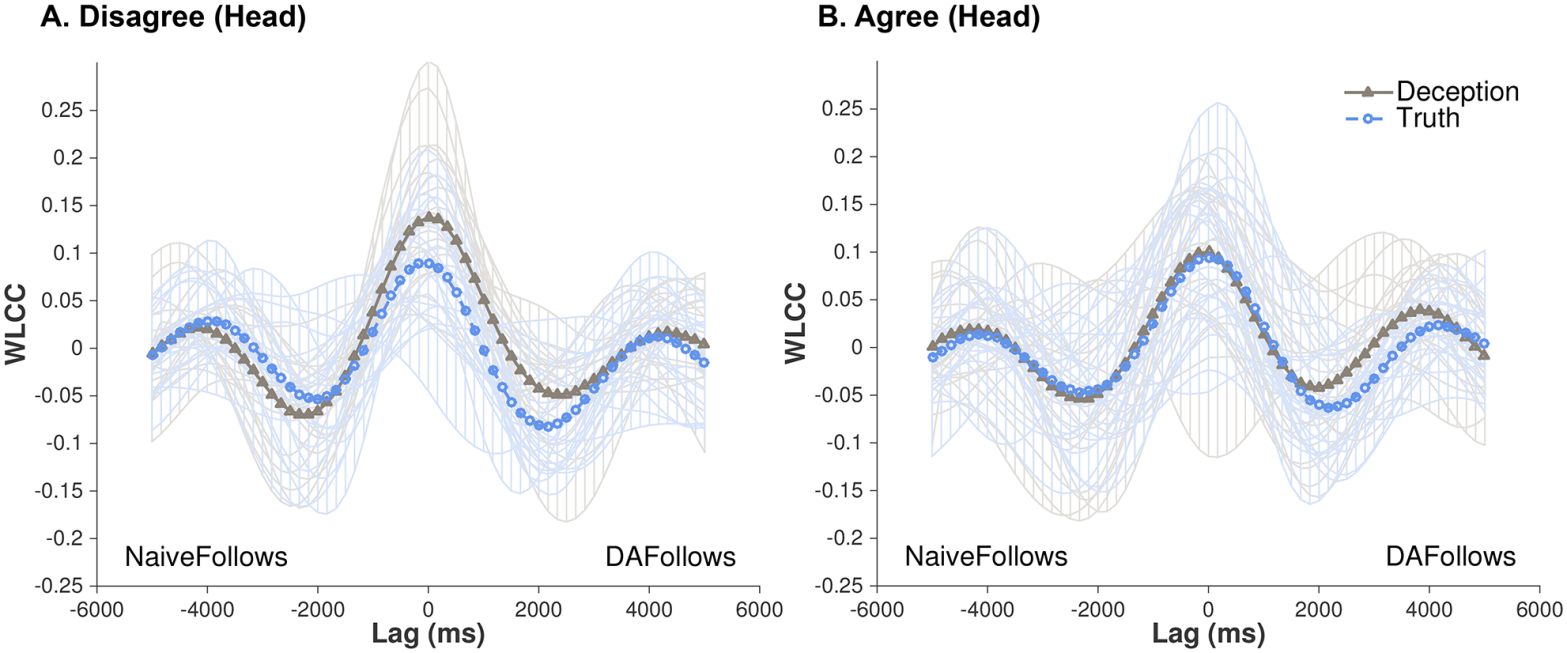
WLCC profiles for actual DAs and naives’ head movements. (A) Disagree and (B) Agree conversations involving deception (triangle-solid line) or truth (circle-dashed line). For (A), systematic patterns of synchronization were found, peaked at near-simultaneous shared activity (Lag 0) and decreases as movements were lagged. Figures also show each dyad’s contributions (opaque lines) to the average pattern across all conditions.

**Fig 4:**
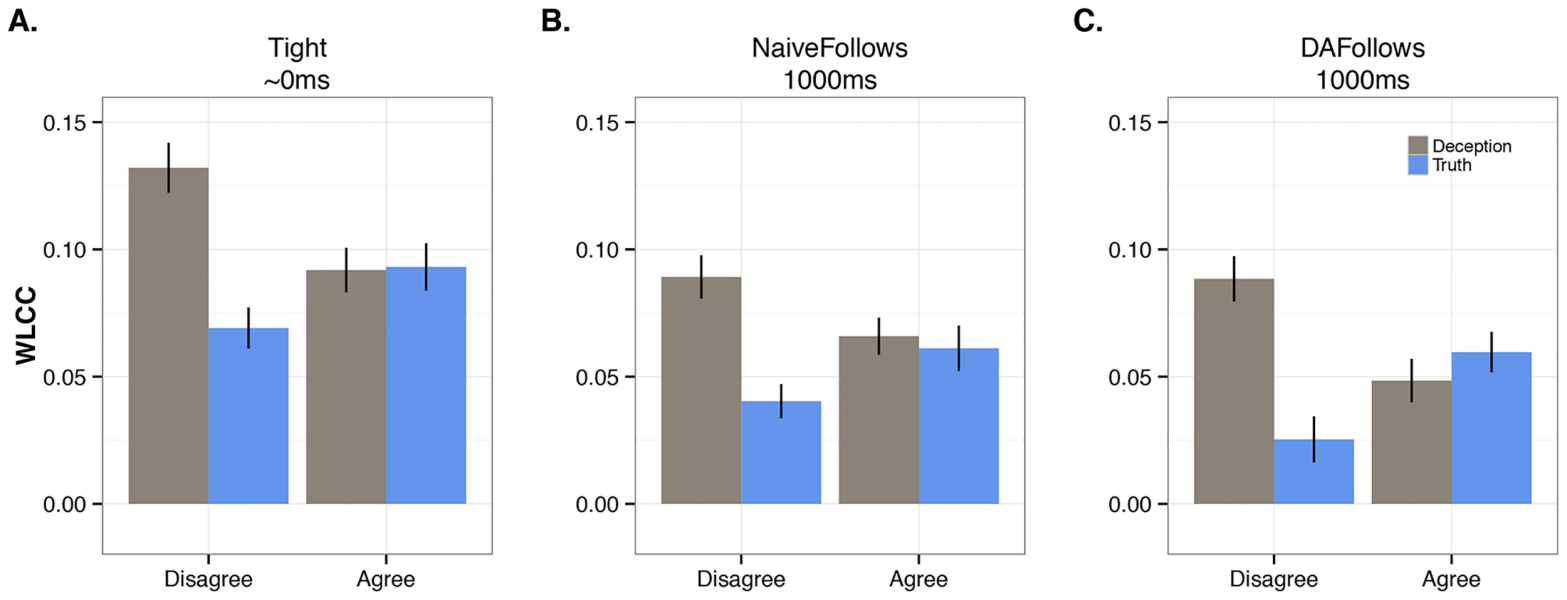
Observed data (mean and standard deviation) of all summary measures for WLCC movement variables. (A) ~0ms Lag, (B) NaiveFollows 1000ms, and (C) DAFollows 1000ms. Each plot compares Veracity (Deception = gray bars; Truth = blue bars) and Conflict conversational conditions.

Fig 3 shows the WLCC profiles for real partners across all conditions, whereas Fig 4 shows a supplementary interpretation of this data as means and standard errors of WLCC scores for Lag ~0ms, NaiveFollows 1000ms, and DAFollows 1000ms. Based on visual comparison, there are noticeable differences between Veracity and Conflict, and indeed, statistical tests reveal critical main effects and interactions between the two conditions (see Table 1). We explore these effects in the following subsections.

**Table 1:** Mixed-effects model results for statistically significant interactions involving Lag ~0ms and DAFollow1000ms. Test results for Veracity condition (Deception versus Truth) within each level of the Conflict condition (Disagree or Agree); and Conflict condition (Disagree versus Truth) within each level of the Veracity condition (Deception or Truth). We report the coefficients associated with the *p*-value, and the standard error of the coefficient.

	Deception Versus Truth		Disagree Versus Agree	
	Disagree	Agree	Deception	Truth
Lag ~0ms	0.064** (0.021)	-0.007 (0.028)	0.099* (0.05)	-0.03* (0.013)
DAFollow1000ms	0.063* (0.024)	-0.009 (0.02)	0.067° (0.034)	-0.037** (0.013)

Note:

** *p* < 0.01,

* *p* < 0.05

#### Lag ~0ms (Fig 4A)

There were no statistically significant main effects for Veracity and Conflict, but there was a critical interaction between these two factors (*Rm*^*2*^ = 0.069, *R*^*2*^ = 0.966, *B* = 0.062, *SE* = 0.027, *p* = 0.021). As the results in Table 1 show, when comparing deception versus truth within each of the Conflict conditions (i.e., holding disagreement or agreement constant), the WLCC values for deception were much greater in disagreement conversations (deception: *M* = 0.132/*SE* = 0.01; truth: *M* = 0.069/*SE* = 0.008; *Rm*^*2*^ = 0.145, *R*^*2*^ = 0.966, *p* = 0.003). For agreement conversations, there were no statistical differences (deception: *M* = 0.092/*SE* = 0.009; truth: *M* = 0.093/*SE* = 0.009). Next, when examining deception alone, but now comparing it across disagreement and agreement conversations, deception in disagreement was higher than in agreement (*M* = 0.132 versus *M* = 0.092; *Rm*^*2*^ = 0.119, *R*^*2*^ = 0.714, *p* = 0.048). Lastly, when examining truth alone, comparing it across disagreement and agreement conversations, the anticipated effect of higher coordination in agreement was found (*M* = 0.093 versus *M* = 0.069; *Rm*^*2*^ = 0.064, *R*^*2*^ = 0.137, *p* = 0.02).

#### NaiveFollows 1000ms (Fig 4B)

There were no statistically significant main effects for Veracity and Conflict, nor was there an interaction between these two factors.

#### DAFollows 1000ms (Fig 4C)

This temporal range followed a similar pattern as Lag ~0ms. Although there were no statistically significant main effects, there was a critical interaction between Veracity and Conflict (*Rm*^*2*^ = 0.053, *R*^*2*^ = 0.844, *B* = 0.07, *SE* = 0.031, *p* = 0.024). Again, as Table 1 indicates, when holding Conflict constant, and comparing deception versus truth, the WLCC values for deception were much higher in disagreement conversations (deception: *M* = 0.088/*SE* = 0.009; truth: *M* = 0.025/*SE* = 0.009; *p* = 0.01, *Rm*^*2*^ = 0.151, *R*^*2*^ = 0.856). For agreement conversations, there were no differences (deception: *M* = 0.048/*SE* = 0.009; truth: *M* = 0.06/*SE* = 0.008). When holding Veracity constant, and comparing deception between disagreement and agreement conversations, deception in disagreement conversations was at its highest (*M* = 0.088 versus *M* = 0.048; *Rm*^*2*^ = 0.088, *R*^*2*^ = 0.577, *p* = 0.05). For conversations that just involved the truth, there was higher coordination in agreement conversations versus disagreement (*M* = 0.06 versus *M* = 0.025; *Rm*^*2*^ = 0.04, *R*^*2*^ = 0.102, p = 0.003).

#### Sway

**Fig 5:**
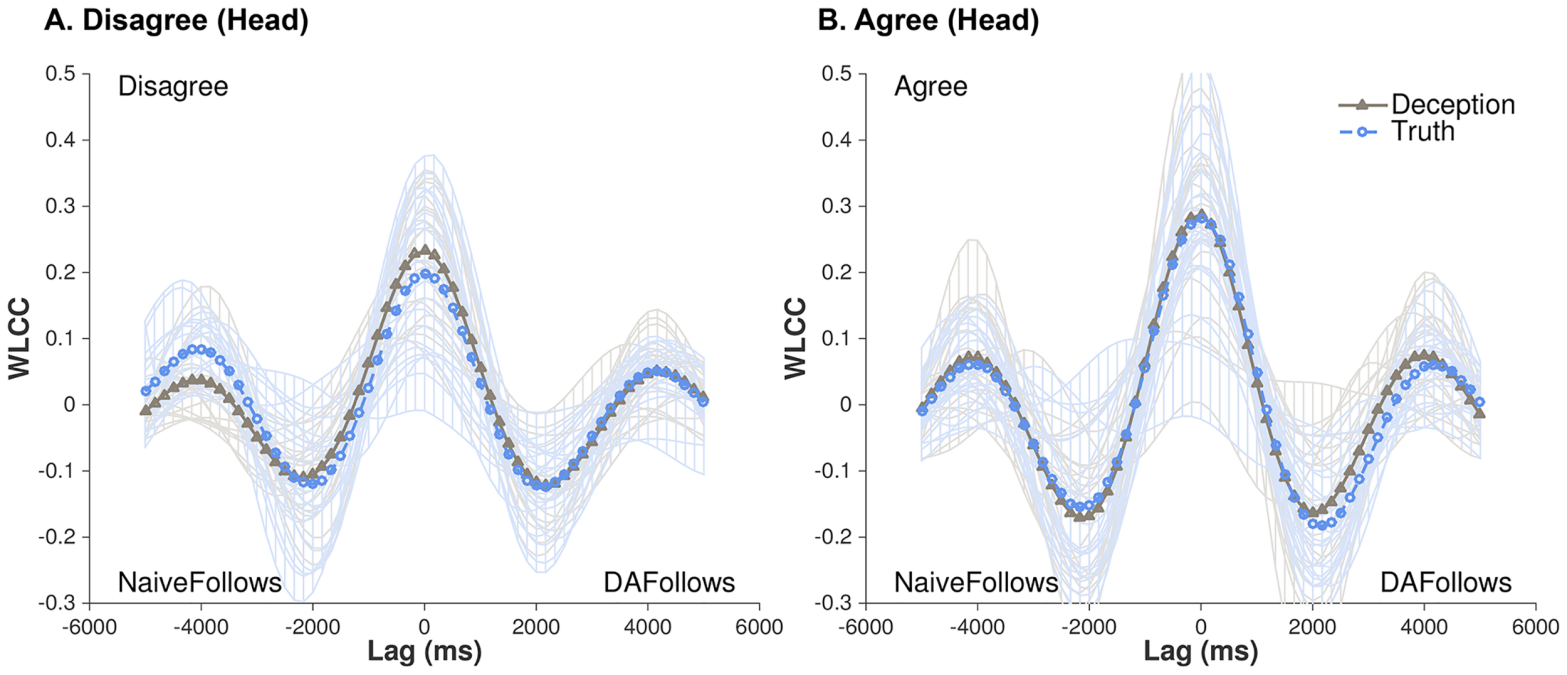
WLCC profiles for DAs and naives’ lower body movements. (A) Disagree and (B) Agree conversations involving deception (triangle-solid line) or truth (circle-dashed line). Although systematic patterns of high synchronization were revealed across all conditions, no statistically significant differences were found. Note: y-axis scaled from -0.30 to 0.50, reflecting the larger cross correlation values for lower body. Figures also show each dyad’s contributions (opaque lines) to the average pattern across all conditions.

We also conducted a follow-up analysis on the movements expressed in the lower body of each participant (from mid-thigh to feet) to ensure that the head movement patterns were not driven by general body sway. Fig 5 shows tightly coupled patterns of coordination for these movements, with cross-correlation values around r = 0.235. However, in contrast with the head movement results, no statistically significant differences were found across conditions or in interaction. There were also no statistically significant effects involving Sex or Order.

## Results: Speech rate coordination

### Virtual pairs

We now examine the patterns of alignment in speech rate coordination, beginning with analyses involving virtual pairs. The conversations across all conditions and for all CRQA measures displayed statistically higher levels and structure of coordination when compared to virtual controls. This result indicates that coordination patterns in the conversation data are due to the way interlocutors adapt to each other and not to the values distribution in the data, or to the structure of conversations and specific conditions (all indexes of recurrence *Rm*^*2*^ and *R*^*2*^ > 0.4, p<0.00001). There were also no statistically significant effects involving Sex or Order.

### Real pairs

**Fig 6:**
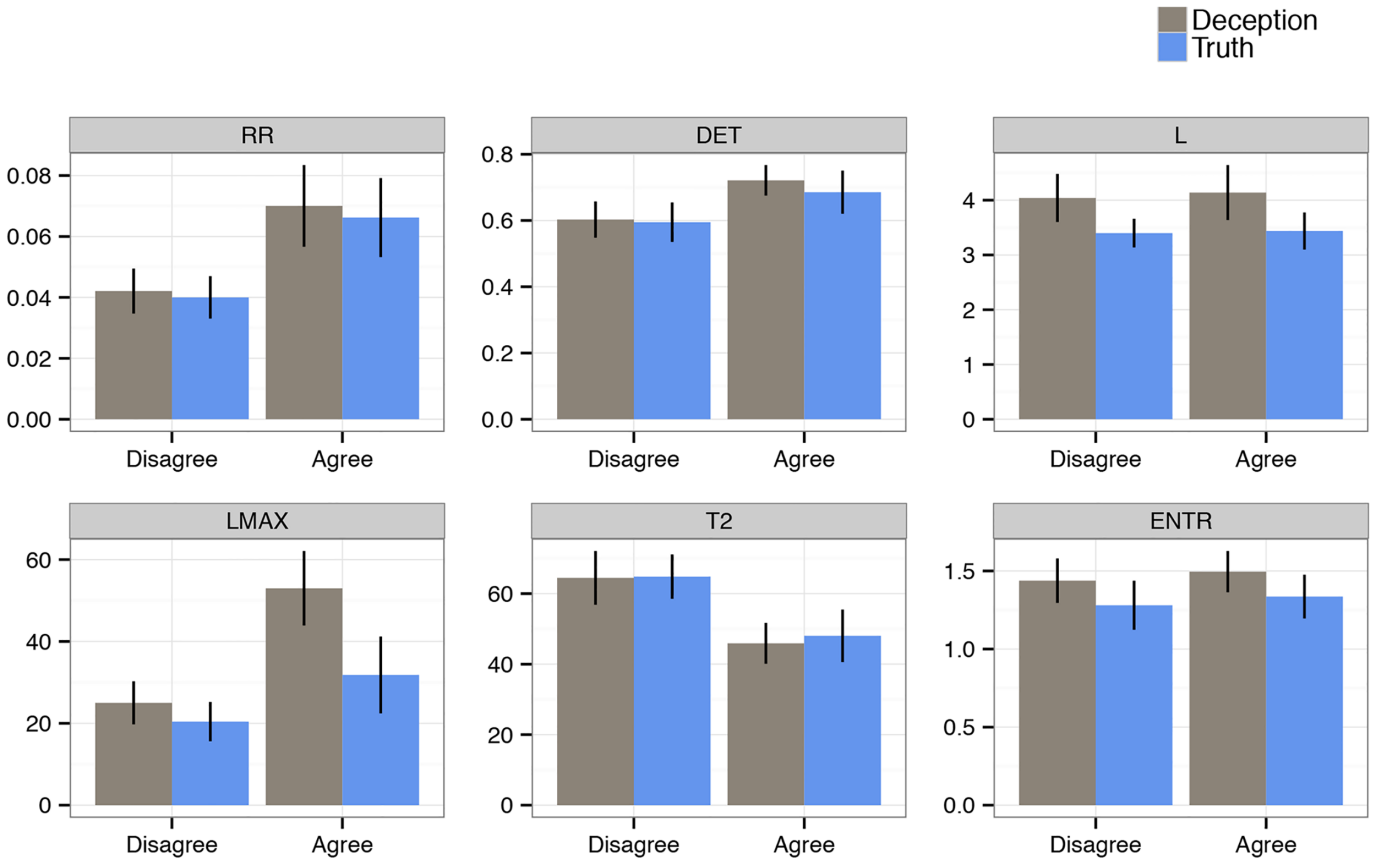
Observed data (mean and standard deviation) of all summary measures for CRQA speech rate variables. Each plot compares Veracity (Deception = gray bars; Truth = blue bars) and Conflict conversational conditions.

The results of the main analyses examining Veracity and Conflict can be seen in Table 2, and the means and standard deviations across conditions for each CRQA measure is plotted in Fig 6. The models revealed statistically significant main effects for Veracity (deceptive vs. truth) and Conflict (disagreement vs. agreement), but no interaction. In deceptive conversations, compared to truth, there were higher values for *L* (deception: *M* = 4.076/*SE* = 0.328, truth: *M* = 3.414/*SE* = 0.203) and *LMAX* (deception: *M* = 35.267/*SE* = 5.251, truth: *M* = 24.893/*SE* = 4.714). Both measures indicate that interlocutors come to share lengthier and more stable patterns of speech rate. Moreover, deceptive conversations also had higher values for *ENT* (deception: *M* = 1.459/*SE* = 0.101, truth: *M* = 1.302/*SE* = 0.108), indicating that patterns of speech rate were not stereotyped (the same throughout the whole conversation). Taking all three measures together, speakers in deceptive conversations can be described as being more adaptively synchronized.

**Table 2:** Mixed-effects model results for CRQA speech rate dependent variables. Dependent variables organized across columns, modelled as a function of the predictors Veracity (Deception, Truth) and Conflict (Disagree, Agree). We report the coefficients associated with the *p*-value, the standard error of the coefficient, and the captured variance as Marginal R^2^ (Rm^2^) and Conditional R^2^ (R^2^).

Variable	RR	DET	L	LMAX	T2	ENTR
**Veracity**	-0.001 (0.007)	0.031 (0.039)	0.616* (0.251)	12.463** (4.386)	-12.339 (9.219)	0.187* (0.093)
**Conflict**	-0.035** (0.012)	-0.178* (0.085)	-0.544 (0.503)	-25.582** (8.503)	35.814* (13.891)	-0.183 (0.195)
Veracity*Conflict	-0.003 (0.011)	-0.002 (0.071)	-0.187 (0.52)	-12.227 (8.931)	-25.877 (13.658)	0.024 (0.184)
**Rm**^**2**^**/R**^**2**^	0.195/ 0.774	0.106/ 0.728	0.105/0.593	0.243/ 0.634	0.16/ 0.655	0.094/0.643

Note:

** *p* < 0.01,

* *p* < 0.05

Next, disagreement conversations, compared to agreement, showed less synchronized speech rate overall, as evidenced by lower values of *RR* (disagree: *M* = 0.041/*SE* = 0.005, agree: *M* = 0.068/*SE* = 0.009). When interlocutors did match speech rate, it was more unstable and not as sustained as that of agreement conversations. This conclusion is based on lower values for *DET* (disagree: *M* = 0.599/*SE* = 0.04, agree: *M* = 0.703/*SE* = 0.039) and *LMAX* (disagree: *M* = 22.833/*SE* = 3.55, agree: *M* = 42.409/*SE* = 6.78), as well as higher values for *T2* (disagree: *M* = 64.631/*SE* = 4.93, agree: *M* = 46.961/*SE* = 4.611).

## Results: Subjective ratings of interactional rapport

**Fig 7:**
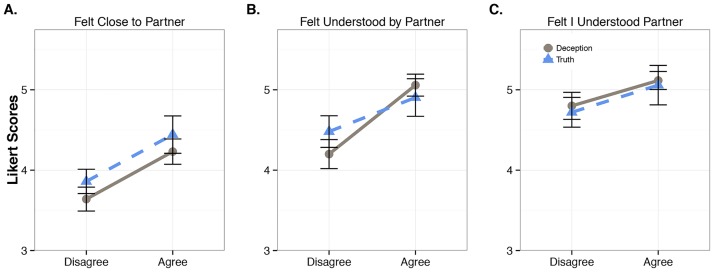
The average Likert-scale ratings of conversational rapport. Values shown on a truncated range (from the original 1 to 6 range) for three questions related to the shared subjective experience of the conversation.

We next consider how the shared subjective experience of the conversations differed across the Veracity and Conflict conditions (see Fig 7; also see S1 and S2 Figs in Supporting Information for a breakdown of DA and naïve participant’s individual subjective experience). There was a statistically significant main effect for Conflict for two of the three follow-up questions. For the "Felt Close to Partner" question, the agreement conversations received a higher rating (disagree: *M* = 3.75/*SE* = 0.106, agree: *M* = 4.337/*SE* = 0.140) (*Rm*^*2*^ = 0.115, *R*^*2*^ = 0.326, *B* = 0.615, *SE* = 0.202, *p* = 0.002), and for the “Felt Understood by Partner" question, the agreement conversations also received a higher rating (disagree: *M* = 4.34/*SE* = 0.134, agree: *M* = 4.981/*SE* = 0.135) (*Rm*^*2*^ = 0.115, *R*^*2*^ = 0.435, *B* = 0.616, *SE* = 0.229, *p* = 0.007). There were no differences reported for deception compared to truth.

The effects above also do not appear to be strongly driven by one partner versus the other. In other words, it does not necessarily follow that the DA consistently rated the conversations as being of lower relational quality than the truth teller. Collapsing across all conditions and questions, the absolute difference score was on average 1.069 (where 5.0 is maximally divergent) with a standard deviation of 1.098. There were also no statistically significant differences when comparing the absolute difference score for each question across conditions (*p* > 0.10 for all comparisons). Although these results fail to reject the null, this is exactly what is expected when the assumption is that meaning and opinion arise at the level of the dyad over the course of extended interaction. At least for the study here with a focus on interaction, participants tend to have largely overlapping assessments.

## Results: Predicting deception and disagreement

Presence of deception was predictable with a balanced accuracy of 66.20% (*CI*: 54.06–77.43, Sensitivity = 68.25%; Specificity = 67.55%), *p* = 0.009, employing solely head movement synchrony at lag -2, with positive coefficients. Analogous results were also achievable employing solely head movement synchrony at lag -3. In other words, the DA following head movement of the naive participant to a lesser degree is a generalizable though weak cue of the presence of deception. Presence of disagreement was predictable with a balanced accuracy of 58.7% (CI: 50.65–67.09%; Sensitivity = 66.02%; Specificity = 56.59%), *p* = 0.02, employing the level of speech rate coordination (*RR* with negative coefficients). In other words, the presence of disagreement is generally revealed by a lower degree of speech rate coordination. For a similar analysis involving the subjective ratings of interactional rapport, we direct the reader to the Supporting Material (S2 File).

## Discussion

In this section, we re-assess our approach to conversational deception and review our results, elaborating on the importance of investigating multiple behaviors, issues of generalizability, and the potential impact these findings may have on the study of deception and interpersonal interactions more generally.

### Dynamical coordination in deceptive interpersonal interaction

Motivated by research on interpersonal interaction, we developed a novel multimodal analysis to examine unique patterns of behavioral coordination in open and extended interactions involving deception. This was done in a novel experimental paradigm whereby participants discussed contentious political topics as pairs who either agreed or disagreed with each other. Crucially, one of the conversational partners was surreptitiously asked to argue an opinion opposite of what was really believed, thus concealing deception from a naïve partner. In this general setup, interpersonal behavioral coordination is conceived as an emergent signal that captures interdependencies between conversational partners as they adapt to the activity at hand and to each other. These interdependencies include the coupling of head movement as local responses over the span of milliseconds, measured via a windowed lagged cross correlation (WLCC) technique, and more global, long-term temporal dependencies in speech rate, captured as structural properties of overall coordination across speech turns using cross recurrence quantification analysis (CRQA). Thus, particular emphasis is placed on immediate and extended forms of coordination, as expressed respectively in head movements and speech rate; aspects that have been underexplored in deception research.

For head movements, the greatest coordination was found in deceptive conversations involving disagreement. This was expressed at moments of tight coupling (~Lag 0ms), and at slightly extended lags when deceivers immediately react to their naive partner (DAFollows 1000ms). These results suggest an immediate and anticipatory responsiveness between deceivers and their conversational partners, with deceivers particularly sensitive to following the lead of their partners and possibly offering more cues for synchronized coordination from the deceived. For shared speech rate dynamics (as syllables per minute), differences between deception and truth, regardless of disagreement or agreement, were found for a key set of speech rate coordination properties. Coordination, over the course of the interaction, can be described as more structured in deception than in truth, such that coordinated speech rate sequences were longer and more stable (L, LMAX), but also more varied (ENT). This combination points to a greater stability but also flexibility in how speech rate coordination was deployed: not stereotyped utterances repeated over and over, but flexible interpersonal adaptation, some times involving short snippets (e.g. backchanneling), sometimes lengthy sequences (full speech acts).

### Deception modulates coordination as an adaptive process

In the introduction, we argued that how interpersonal coordination unfolds during deceptive conversations is very much an open question. On the one hand, coordination is often associated with single, predominant functional explanations involving rapport-building or common ground formation, and as such, deception, particularly deception in a more demanding situation involving disagreement, should be disruptive to stable synchrony. On the other hand, deception presents a unique situation where an overriding and salient goal for the deceiver is to maintain consistency in believability while delivering information known to be false. It was hypothesized that this might instead result in greater attunement to the partner and conversation, particularly in disagreement contexts where being believed might be most threatened. The results reported here provide support for this latter view—a view consistent with a synergistic account where low-level behavioral synchrony is sensitive to a wide-array of contextually relevant intentional goals. This interpretation is also commensurate with research on behavioral mimicry where many of the same moderators on outcome are likely to be similar [14]. There are a number of findings in this domain to show that context-dependent factors and social goals can amplify or diminish mimicry [26, 70]. For example, nonconscious mimicry tends to increase when people’s affilation goals are enhanced [70], or when one’s social relationship is threatened [71]. Such factors are likely relevant to deception and disagreement, where socially-desirable outcomes are important but particular attention needs to be made to establish and maintain them.

Interestingly, when differences between truthful disagreement and agreement conversations were examined, that is, in conversations that did not involve deception, our results were also consistent with previous findings that have shown that body movement coordination increases during agreement (e.g., [45, 72]). We extend these findings to greater head movement and speech rate coordination, as well as to a speech rate coordination that was more stable and sustained for longer periods of time. Moreover, whereas in the deception conversations there was no relationship between self-reported measures of rapport and coordination, in the truth conversations, participants reported greater rapport during agreement relative to disagreement. This suggests that in contexts that do not involve deception, the functional relationship between synchrony and affiliation may be the most clear cut.

Future work will also have to more precisely investigate the exact mechanisms behind interpersonal coordination, rapport, and need for believability in a variety of deceptive contexts. This includes contexts where participants are able to choose when to lie. Although this sort of unsanctioned deception is more commonly implemented in experimental paradigms where opportunities to lie occur at discrete points in a structured event sequence [73–76], there have been recent attempts to capture unsanctioned deception in more naturalistic and open social interactions [12, 77–78]. It is here where concerns about reputation and impression management might be most pronounced [79–80], and where our results might find greater support. Or, as some have argued [81], only the most confident and natural liars take advantage of unsanctioned opportunities for deception, thereby possibly resulting in entirely new patterns of behavior.

Another question worth pursuing is to what extent deceivers strategically increase interpersonal coordination versus its spontaneous emergence due to a greater general attentiveness and monitoring of a conversational partner (also see [82]). Although we suspect the latter given the particular timescale and properties of coordination being assessed here, it could be that strategic and emergent forms of coordination mutually interact.

An additional avenue of future work will be to examine head movements in greater detail, as it was this behavioral signal where the greatest coordination was shown for deception and disagreement—and where the strongest support for the adaptive, synergistic account is made. The relevance of head movements is that they serve direct communicative goals. For example, listeners’ attention is mostly drawn to the speaker’s head and face during conversation [83]. Head movements are also particularly sensitive to conversational demands. A vast repertoire of meaning is conveyed in head movements, from signaling understanding and requests for information, to transitions between discourse topics and making lexical repairs [84]. However, the motion we tracked does collapse over these discrete categories to produce a simple rhythm of continuous change in overall displacement. This presents a disadvantage in that we are unable to make clear connections between local conversational functions (e.g., emphasis, agreement) and specific behaviors (e.g., nodding up and down, shaking side to side). Doing so will be relevant when taking a more granular focus on how momentary conversational goals and speech acts relate to changes in coordination. But this is not to say that there are not notable advantages using a frame-by-frame motion extract technique as done here. The resulting motion energy flows used here have been shown to carry a wealth of information about others’ mental states [85, 32]. Moreover, as the basis for generating a signal of tightly coupled coordination, it cannot be easily manipulated by skilled deceivers in the same way that specific, individualized behaviors can. With further attempts to explore even finer-grained continuous changes in movement, we expect that even more pronounced patterns in coordination might be revealed. Indeed, recent research using infrared motion tracking cameras is uncovering such differences [77].

### Timescales of synchrony

One aspect of our research that sets it apart from related work is in the examination of multiple types of coordination using the statistical methods of WLCC and CRQA across various temporal and spatial scales. With WLCC, for example, locally coupled sequences of shared head movements across minimal delays in time (less than 1000ms) were found to be highly synchronized. This result is notable because traditional information transmission models assume the necessity of a delay between perceiving a behavior and reacting to it, as there is a need to decode and encode information. With little evidence of such a delay, our results support the idea of “ultrafast” synergetic cognition [59]. In other words, the conversational partners’ responsiveness to each other is highly anticipatory, allowing for the rapid alignment of speakers/listener into a single functional unit [18, 86–88]. Again, the fact that this was most evident in deception conversations involving disagreement underscores the greater attunement between conversational partners that may have been particularly pronounced.

For speech rate adaptation, we were interested in coordination that occurs as long-term temporal dependencies across the entire interaction. By using CRQA, we could examine coordination that is not limited to contiguous sequences of behavior—a behavior in speech turn-taking that does not easily allow for such analysis given it alternates between individuals (one person is speaking while the other is not). CRQA bypasses this problem, and allows one to capture overall synchrony, as well as global properties of stability and complexity in coordination. This leads to potentially new insights, such as our finding that deception, relative to truth, showed properties of more stable yet flexible patterns of synchrony. This combination aligns well with notions that deception requires the deceiver to maintain consistency in believability while nimbly responding to suspicion [77, 89].

The CRQA results also raise the issue of how much the speech rate coordination driven is driven by particular linguistic behaviors, such as the repetition of words or syntactic structures. An important finding in previous research is that during conversation, interlocutors’ language can converge and become more similar in systematic ways [90–91]. This convergence helps explain the ease and speed in which people create, express, and maintain common ground in conversation [15, 92], and also has been shown to be modulated by perspective-taking and socially-desirable outcomes that are similar to those involved in deception [93–95]. Although the current results do not directly address the relationship between linguistic convergence and speech rate coordination, our data does allow such questions to be asked, and is indeed a current focus of ongoing research [96].

### Generalizability and prediction

Deception researchers have long been interested in identifying behavioral cues that are most associated with deception. Although discriminant function analysis and other regression-based techniques have proven useful in identifying salient cues, there is still room for improvement by integrating these techniques with methods that also account for greater generalization. For a field where detection accuracy is central, this more rigorous testing should be welcomed, but is not always done.

We introduced a cross-validated machine learning approach to identify non-redundant and predictive cues from 20 potential independent variables. In doing so, only one behavioral variable was revealed (e.g., a nuanced measure of lagged head movements), suggesting that behavioral channels, across multiple modalities, might share similar dynamics (redundant to a degree). With this variable, and using cross-validated regression models, we also achieved a deception prediction rate of 66.20%. This rate is not too far away from other studies using automated motion extraction techniques (e.g., low 70% range; [97]). Even so, the prediction results, at least at the present moment, suggest more needs to be done to achieve acceptable prediction rates for a forensic context (a perennial and unsolved problem in detection research), for instance by integrating cues from individual behaviors and interpersonal patterns and by exploring additional behavioral channels.

From a theoretical perspective, however, there are potentially valuable insights to be gained. It seems that the behavior expressed from dyad to dyad is highly variable, and thus hinders the ability to generalize to new dyads. In contrast to our mixed effects results, where multiple behavioral cues were associated with deceptive conversations, cross-validation intentionally does not control for idiosyncratic differences to assess whether the results would generalize to new never-seen-before participants. The weaker results reinforce the notion that deceptive behaviors are better understood as a within-participant (-dyad) phenomenon, at least when expressed in naturalistic, open-ended, and extended interactional contexts.

### Conclusions

This study proposed a novel experimental paradigm (the Devil’s Advocate) to investigate deception and conflict in conversation. We employed advanced time-sensitive methods to quantify interpersonal coordination (coupling forged in tight perception-action loops and longer-term global properties) at multiple behavioral levels (head movement and speech rate). The findings highlight the traces that deception and conflict leave on interpersonal dynamics, showing that such dynamics can be described as specific modalities of multimodal interpersonal engagement (or synergy). More specifically, the findings show that coordination is not simply a function of rapport and reciprocal understanding, but multiple factors play a role: e.g. high-level goals, reciprocal attention, and attunement. In other words, low-level coordination can be shaped by a number of high-level intentions and communicative constraints. The study paves the way for further investigations of conversation and coordination within a less savory, but definitely more realistic, intermeshing of attunement, conflict, and deception. Within the field of deception studies, it highlights the need for more fine-grained and multimodal analyses of deception in extending our understanding of the interplay between individual and interpersonal dynamics.

## Supporting information

S1 File Github resources. (DOCX)

S2 File Predicting deception and disagreement with subjective ratings. (DOCX)

S1 Fig *Naive participant only*: The average Likert-scale ratings of conversational rapport. Values shown on a truncated range (from the original 1 to 6 range) for three questions related to the naïve participant’s subjective experience of the conversation.
(DOCX)

S2 Fig *DA participant only*: The average Likert-scale ratings of conversational rapport. Values shown on a truncated range (from the original 1 to 6 range) for three questions related to the DA participant’s subjective experience of the conversation.
(DOCX)
