# Supplementary material for: Conversing with a devil’s advocate: Interpersonal coordination in deception and disagreement
Source: PLoS One. 2017 Jun 2;12(6):e0178140. doi: 10.1371/journal.pone.0178140 (PMC5456047; doi:10.1371/journal.pone.0178140)
Supplement: S1 File — (DOCX) [file pone.0178140.s001.docx]

Please note that additional data files and code referenced in the main manuscript can be found at <https://github.com/nickduran/coordination-deception>, or can be directly requested by contacting Nicholas Duran at [Nicholas.Duran@asu.edu](mailto:Nicholas.Duran@asu.edu).
