# Supplementary material for: Conversing with a devil’s advocate: Interpersonal coordination in deception and disagreement
Source: PLoS One. 2017 Jun 2;12(6):e0178140. doi: 10.1371/journal.pone.0178140 (PMC5456047; doi:10.1371/journal.pone.0178140)
Supplement: S2 File — (DOCX) [file pone.0178140.s002.docx]

Here we ask the question of whether synchronization indexes contribute additional information beyond experimental manipulations in “predicting” rapport? In other words, we extended the analysis reported in the main manuscript under the heading “Results: Predicting Deception and Disagreement” to include predictions of rapport. We found that Speech Rate Recurrence Rate (RR) and Head Synchronization at Lag -1000ms both increase the explanatory power of our models (beyond “Conflict” and “Deception” variables) for the feeling of having understood one’s interlocutor, but not Feelings of Closeness or of Being Understood. Note: Betas are z-scored.

- Speech Rate RR: Beta=0.31, SE=0.13, t=2.39, p=0.03
- Head Synchronization at Lag -1 second: Beta=-0.22, SE=0.09, t=-2.47, p=0.015
