# Supplementary material for: Conversing with a devil’s advocate: Interpersonal coordination in deception and disagreement
Source: PLoS One. 2017 Jun 2;12(6):e0178140. doi: 10.1371/journal.pone.0178140 (PMC5456047; doi:10.1371/journal.pone.0178140)
Supplement: Supplementary file 4 [file pone.0178140.s004.docx]

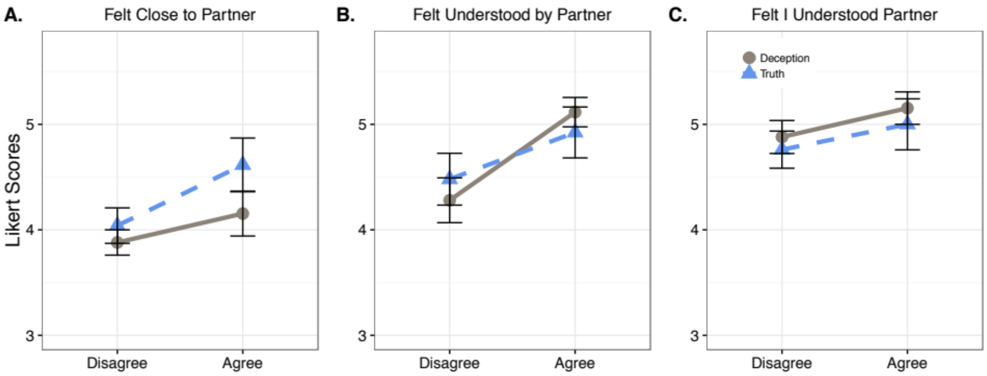

A.
Felt Close to Partner
B.
Felt Understood by Partner
C.
Felt I Understood Partner
Deception
Truth
Likert Scores
5
4
3
Disagree
Agree
